# Circulating Molecular Biomarkers for the Diagnosis and Monitoring of NSCLC—A Review

**DOI:** 10.3390/ijms262110278

**Published:** 2025-10-22

**Authors:** Wojciech Jelski, Sylwia Okrasinska, Weronika Rutkowska, Barbara Mroczko

**Affiliations:** 1 Department of Biochemical Diagnostics, Medical University, 15-269 Bialystok, Poland; mroczko@umb.edu.pl; 2 Department of Biochemical Diagnostics, University Hospital, 15-269 Bialystok, Poland; sylwia.okrasinska@uskwb.pl (S.O.); weronika.rutkowska@uskwb.pl (W.R.); 3 Department of Neurodegeneration Diagnostics, Medical University, 15-269 Bialystok, Poland

**Keywords:** tumor biomarkers, non-small cell lung cancer

## Abstract

Non-small cell lung cancer (NSCLC) is a severe disease with a very poor prognosis. Some 30–80% of patients with NSCLC die within five years of cancer diagnosis. The main factors contributing to this condition are the lack of effective markers for diagnosing cancer at an early stage, as well as the complexity of the biological processes involved in tumorigenesis and progression. The development of knowledge regarding all aspects of NSCLC has provided information used in the detection, systemic anticancer therapy and monitoring of NSCLC, which has a significant impact on prognosis and quality of life. NSCLCs release various biological substances into the bloodstream. Liquid biopsies allow for the analysis of tumor components in body fluids, and the usefulness of these biopsy tests as a substitute for tumor tissue is increasing. In this article, we critically review the available literature on microRNAs, circulating cell-free DNA (cfDNA), tumor-educated platelets (TEPs), circulating tumor cells (CTCs), circulating extracellular vesicles (EVs), and metabolomic and proteomic markers in the diagnosis and monitoring of NSCLC. However, the usefulness of these new markers in clinical practice has significant limitations.

## 1. Introduction

**Figure 1:**
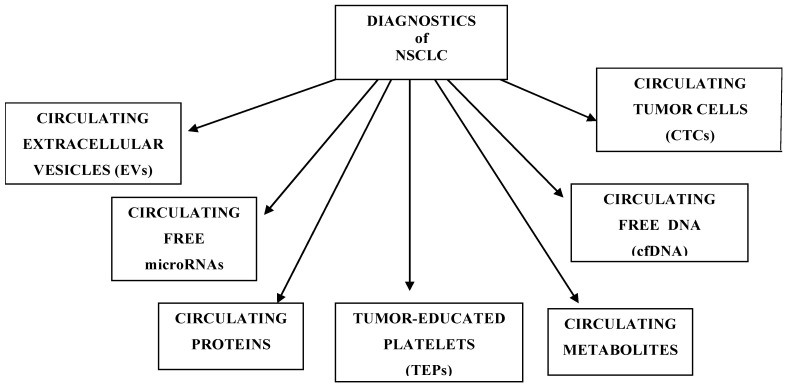
Division of NSCLC biomarkers.

**Figure 2:**
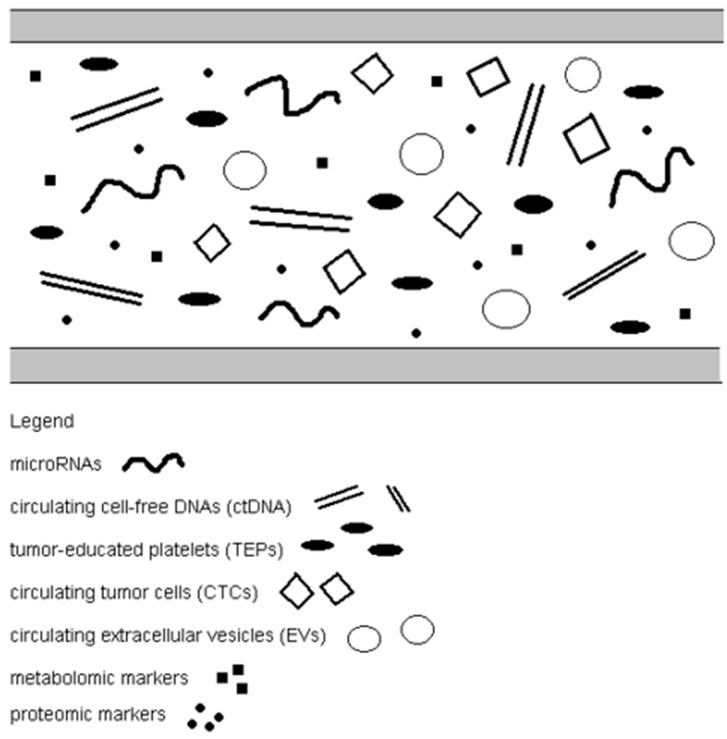
Liquid biopsy materials in NSCLC.

Lung cancer is the most common human cancer worldwide, including in the US, and a leading cause of mortality (accounting for approximately 19% of cancer deaths). The incidence of lung cancer varies by gender (men are more likely to be ill) and age (over 45% of patients are >70 years old) [1]. The overall five-year survival rate of patients with lung cancer does not exceed 20%, mainly because the cancer is diagnosed at a late stage. Unfortunately, lung tumors remain asymptomatic for a long time, infiltrate surrounding tissues, and metastasize to distant organs. It is crucial to include as much of the general population as possible in X-ray screening to detect lung cancer at the earliest stages. It is also important to find better technologies that will enable early detection of cancerous lesions in the lungs and increase survival rates for lung cancer. Among lung cancers, non-small-cell lung cancer (NSCLC) is the most common type (85%) [2]. The basic treatment for stage 1 NSCLC is tumor resection. However, almost half of patients experience recurrences despite resection, mainly due to distant metastases [3]. Thus, it is necessary to better understand the molecular mechanisms of non-small cell lung cancer progression in order to develop modern diagnostic methods for early diagnosis and prognosis of NSCLC. The gold standard for diagnosing NSCLC is tissue biopsy. However, the method based on these biopsies has limitations, including invasiveness, the potential risk of failure to detect early stages of cancer or small changes, and insufficient possibility to monitor therapy and carry out prognostic assessment. Tumor biopsy is often dangerous due to the anatomical location of the primary cancer or metastases as well as the deteriorating general condition of the patient [4]. Patients’ chances of survival and recovery depend on the stage of disease progression at the time of detection, which is why new markers that enable early tumor diagnosis are crucial. Molecular biomarkers present in the blood, known as liquid biopsies, represent a second option or complement to tumor biopsy and imaging studies, enabling better characterization of the tumor’s molecular factors and response to treatment in a non-invasive and easily repeatable manner. Liquid biopsy is a cytological and molecular analysis of various substances, compounds, and cell fragments secreted by the tumor into bodily fluids. Liquid biopsy has the potential to revolutionize oncology by addressing several limitations of traditional tissue biopsies. This innovative, non-invasive technique has enormous potential to optimize the treatment of non-small cell lung cancer. It could be a valuable diagnostic tool for identifying molecular alterations that can be treated pharmacologically. New applications could include early detection, real-time monitoring, and assessment of spatial and temporal heterogeneity in NSCLC. Analysis of liquid biopsy diagnostics in early-stage NSCLC (stage I IIIA/locally advanced/low stage) shows sensitivity of around 0.27 (95% CI: 0.14 0.46) for detecting tumour-derived mutations via ctDNA in low-stage disease; in contrast, high-stage disease sensitivity is much higher (~0.75). For example, diagnostic biomarkers (ctDNA, methylation, miRNA, etc.) in early-stage non-small cell lung cancer give an AUC of ~0.85. Liquid biopsy, as a rapidly developing method of cancer research, may be useful at various stages of doctors’ procedures: early diagnosis, therapy monitoring, and assessment of the risk of recurrence and disease progression. The most common material used in liquid biopsy is blood because it provides the widest range of biological analytes, such as microRNAs, circulating cell-free DNA (cfDNA), tumor-educated platelets (TEPs), circulating tumor cells (CTCs), circulating extracellular vesicles (EVs), and metabolomic and proteomic markers (Table 1, Figure 1 and Figure 2) [5].

Technical Roadmap for NSCLC Liquid Biopsy

1. Pre-Analytical Phase
  - Blood Collection:
  Specialized blood collection tubes are used to stabilize nucleic acids and cells, for example
    - CTCs: CellSave Preservative Tubes (specific to the CellSearch system)
    - RNA: PAXgene Blood RNA Tubes (for RNA stabilization)
  - Sample Handling:
  Samples are maintained at room temperature (for Streck tubes) or on ice (for EDTA)
  - Centrifugation Protocol:
  Step 1: Whole blood is centrifuged at 1600× *g* for 10 min at 4 °C to separate plasma
  Step 2: Plasma is transferred carefully without disturbing buffy coat
  Step 3: Second centrifugation at 16,000× *g* for 10 min at 4 °C to remove residual cells/debris
  - Storage: Store plasma at −80 °C until extraction
2. Analytical Platforms
  - Circulating Tumor Cells (CTCs): Platform: CellSearch System (FDA-approved)
  - Cell-Free DNA (cfDNA):
  Detection/Quantification:
    - ddPCR (Droplet Digital PCR): Ultra-sensitive mutation detection (e.g., EGFR T790M)
    - NGS (Next-Generation Sequencing): Comprehensive mutation profiling
  - Cell-Free RNA (cfRNA)/RNA Profiling
    - NanoString nCounter System: Multiplexed RNA expression profiling (gene signatures, immune profiling)
    - qRT-PCR: Targeted RNA quantification (e.g., fusion transcripts)
  - Exosomal Analysis (optional):
  Exosome isolation by ultracentrifugation or commercial kits
  Downstream analysis by ddPCR, NGS, or NanoString

## 2. Materials and Methods

**Figure 3:**
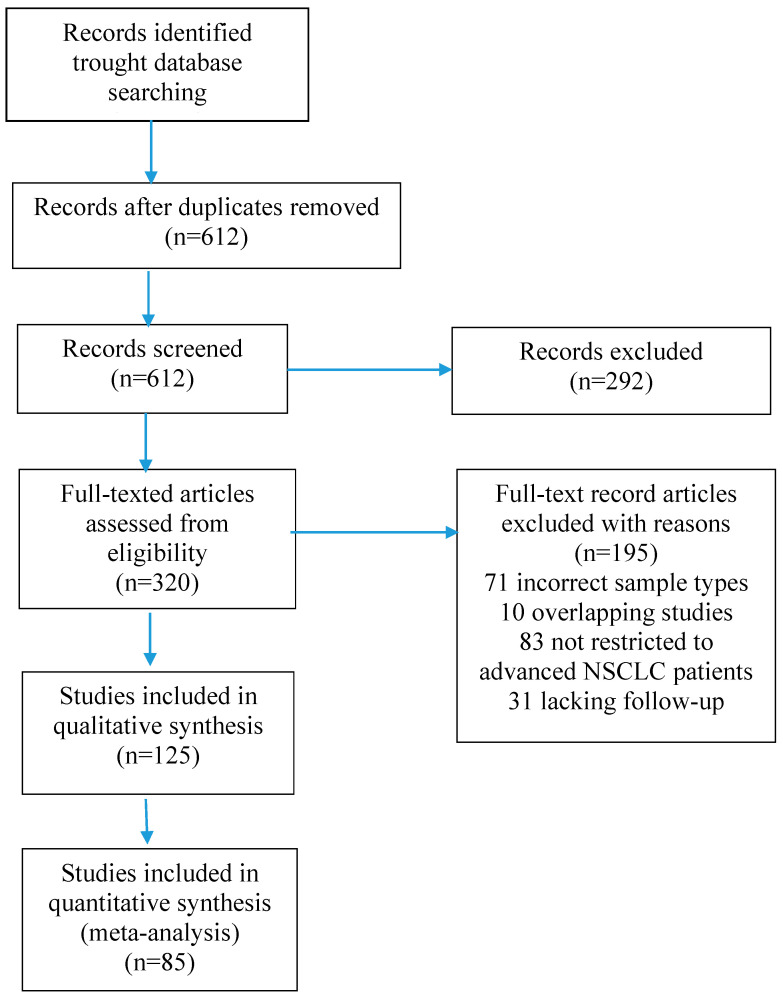
Flow diagram of the selection of studies for the present meta-analysis.

In this systematic review, we comprehensively assessed the diagnostic utility of different biomarkers of NSCLC. The authors conducted an in-depth online search of articles from three databases: PubMed, Embase, and Web of Science. The articles came from the years 2010–2025, with a main focus on the last 5 years. The compilation of these databases yielded satisfactory results, achieving an overall recovery rate of over 90%. Finally, 85 published reviews were included, resulting in a total of 1520 relevant references identified by searching our databases (Figure 3). The subject terms used in the search were “non-small cell lung cancer”, “microRNA”, “circulating tumor DNA”, “circulating tumor cells”, “tumor-educated platelets”, “circulating exosomes”, “metabolomic and proteomic markers” and “diagnostic utility”. We adopted the following inclusion criterion for eligible articles that were analyzed by our full-text assessment: articles regarding the association between the concentrations of the studied biomarkers and the diagnosis of NSCLC. Studies were considered eligible if they met the following criteria: diagnostic ability of a specific biomarker to detect non-small cell lung cancer was reported, and all cancers were diagnosed by histological examination. The exclusion criteria included publications not in English; studies not on humans; reviews, letters, and meeting records; insufficient data; and studies in which NSCLC and the markers of interest were not studied.

## 3. Circulating Tumor Cells

Circulating tumor cells (CTCs) are cells from primary or metastatic cancers that enter different body fluids, including blood, urine, or cerebrospinal fluid, depending on the neoplastic lesions. CTCs play an important role in the development of the metastasis process and determine the migration of cancer cells to secondary sites via the circulatory and lymphatic systems [6]. CTCs are observed as single free cells, clusters of several cells, or circulating large tumor agglomerates that are composed of a large number of cells associated with other cells such as hematopoietic cells, stromal cells, and platelets [7]. Although it has been stated that CTCs play a significant role in the development of metastatic tumor foci, the mechanisms that allow circulating cancer cells to enter the blood and colonize other organs to form secondary foci are very complex and have not been fully explained. Despite their relatively small amounts in various fluids (less than 10 in 10 mL of blood), they are easily available for testing by simple and non-invasive collection of body fluid samples. CTCs are characterized by positive expression of epithelial markers such as epithelial cell adhesion molecules (EpCAMs) and cytokeratins (CKs). They are also negatively expressed by the white blood cell-specific marker CD45. When entering blood vessels, circulating tumor cells undergo epithelial–mesenchymal transformation, and when extravasating to secondary organs, they undergo mesenchymal–epithelial transformation [8]. Therefore, circulating tumor cells in the bloodstream show heterogeneous biomarker expression, depending on epithelial–mesenchymal and mesenchymal–epithelial transformation. Among heterogeneous CTCs, we distinguish three subgroups: CTC clusters, intact single CTCs, and apoptotic CTCs [9]. Circulating tumor cells are composed of cellular and subcellular components useful for further studies, e.g., intact DNA for the analysis of various mutations and the discovery of new markers, RNA for the assessment of gene expression, and different biomarkers for proteomic studies.

A lot of studies have found that CTCs are effective biomarkers for detecting and predicting the prognosis of different cancers, e.g., breast tumor or prostate cancer and colorectal cancer [8,10]. The presence of circulating tumor cells has been detected in the plasma of people with different cancer and correlated with poor prognosis in patients with metastatic non-small cell lung cancer [11]. It is important that CTCs can act as a substitute for tumor tissue and be analyzed for its presence. Observations based on annual computed tomography imaging revealed the presence of lung nodules from one to four years after detection of circulating tumor cells, and CTCs were undetectable after surgery. Studies conducted by Chinese scientists have shown that in vivo CTC detection is characterized by over 50% sensitivity and about 90% specificity in diagnosing early-stage non-small cell lung cancer [12]. Unfortunately, the study group was small, and for this reason, the investigation was insufficient and inconclusive. Additionally, another prospective study conducted on 614 patients with chronic obstructive pulmonary disease hospitalized in 21 French universities observed only 26% sensitivity of circulating tumor cell detection in non-small cell lung cancer, which questions the usefulness of this method in screening NSCLC [13]. In non-small cell lung cancer, CTCs have greater prognostic value that has been extensively studied. The amount of CTCs correlates with the burden of non-small cell lung cancer, and the detection rate has a wide range from 15% to 100% [14,15,16]. Therefore, the utility of CTC counts is greatest in advanced stages of cancer. A decrease in CTC counts observed after therapy suggests tumor remission, while an increase may indicate progression. Changes in the amount of CTCs are associated with shorter disease-free survival, but the optimal level of CTC detection remains unclear [17,18,19]. Shishido et al. in a prospective analysis of stage IV NSCLC identified CTCs in most of the patients studied [20]. Prognosis was not influenced by the absolute CTC count, whereas changes in CTC counts influenced overall survival. The largest study of CTCs in advanced non-small cell lung cancer involved 550 people in a multicenter study across Europe. Researchers confirmed that CTCs have independent prognostic value for overall survival [21]. The detection of circulating tumor cells in combination with imaging and clinical data has greatly improved diagnostic accuracy in early-stage NSCLC [22]. Similar data have been reported for small cell lung cancer. Analysis of CTCs levels in patients with limited-stage small cell lung cancer who received concurrent chemoradiotherapy in the CONVERT trial demonstrated that a threshold of ≥15 CTCs was associated with shorter survival time independent of all other factors [23]. For many years, there has been a consensus that CTCs correlate with tumor aggressiveness and have been studied as a surrogate marker for tumor growth. It has been shown that a decrease in the number of circulating tumor cells during therapy is associated with radiographic tumor response and an increase in cell counts with tumor growth [24]. It has also been shown that the reduction or disappearance of CTCs as a result of surgical resection correlates with better clinical outcomes [25]. Bayarri-Lara C et al. prospectively analyzed the kinetics of CTCs in the blood of 56 patients collected before and one month after surgery. They found that the presence of CTCs after surgery was significantly associated with early recurrence and shorter disease-free survival [26]. A telomerase-based CTC assay in patients with early-stage NSCLC who were treated with stereotactic body radiotherapy showed that higher pretreatment CTCs (more than five cells per milliliter) and persistent CTCs after a treatment cycle indicated an increased risk of recurrence locally or in another organ. This test enables the identification of patients who would benefit greatly from adjuvant systemic treatment and allows for early diagnosis of relapse or progression [27].

Despite technological advancement, circulating tumor cells are currently not used in everyday diagnostic practice, nor do they constitute material for biomarker analysis in advanced NSCLC. Unfortunately, there are limitations that make CTC detection difficult. These include the lack of epithelial biomarkers, insufficient validation in numerous population-based studies estimating the level of diagnostic value parameters, and the low level of clinical validation in individual cohorts of lung cancer patients. Therefore, additional studies are needed in advanced NSCLC to implement CTC labeling and its use in clinical settings (Table 2).

## 4. Circulating Free miRNA

A separate and interesting category of molecular biomarkers for NSCLC are extracellular microRNAs (miRNAs), which are highly stable even in unfavorable conditions (Table 3). The level of microRNAs can be determined in plasma, serum, or alveolar lavage fluid. This is a class of small non-coding RNAs whose mature products are approximately 20 nucleotides long. They influence the regulation of gene expression at the post-transcriptional and/or translational stages. MicroRNAs play a crucial role in maintaining balance and intercellular communication in both healthy and cancer cells. MicroRNAs participate in development of cancer by influencing growth, apoptosis, and differentiation. Analysis of microRNAs has allowed for the precise identification of the origin of neoplastic tissue in various cancers [28].

Changes in blood microRNA concentrations may correspond to various mechanisms of biological processes occurring in cancer. The source of microRNAs released into plasma in NSCLC patients are not only the primary tumor, CTCs, micrometastases, and pre-cancerous tissues in other organs; they may also originate from stimulated neighboring cells. More than half of patients with non-small cell lung cancer who had no clinical evidence of distant metastases or nodules had evidence of hematogenous dissemination of cancer cells. These early disseminated cancer cells can colonize distant organs and survive for several years as dormant cells in patients without any clinical evidence of metastases [29,30,31]. This can be considered a systemic response to cancer, involving the development of cells from other tissues, including platelets, leukocytes, and erythrocytes, within the tumor. Among other things, it has been found that whole blood cells in people with NSCLC exhibit deregulated microRNA expression. In patients with NSCLC, the levels of some deregulated microRNAs in the blood before surgery decreased to levels comparable to those in the group of patients after tumor resection. This normalization as a result of resection suggests that the abnormal levels of these microRNAs in the plasma are influenced by the presence of the primary tumor and may have potential as a biomarker for NSCLC [32].

Some microRNAs have been found to be an independent prognostic factors. These microRNAs include microRNA-21-5p, microRNA-32-5p, microRNA-502b-3p, microRNA-150-5p, microRNA-122-3p, and microRNA-92a-3p. Of particular interest is plasma microRNA-32-5p, which was elevated preoperatively in patients with relapse, whereas it was decreased in those without relapse. Of note, a recent study suggests a tumor suppressor role for this microRNA in NSCLC [33]. Studies of patients with early-stage non-small cell lung cancer showed a sensitivity exceeding 80% for microRNA-20a, microRNA-2223, microRNA-145, and microRNA-448. Specificity exceeding 90% was observed for microRNA-628-3p, microRNA-210, microRNA-29c, and microRNA-1244 [34]. In a cohort study of over 3000 patients, the microRNA panel identified patients with lung cancer with approximately 91% accuracy, greater than 82% sensitivity, and 93.5% specificity. However, the group included patients with a wide variety of other conditions, both pulmonary and non-pulmonary [35]. Ulivi et al. assessed the prognostic value of microRNAs in a study of 192 patients with NSCLC (99 with adenocarcinoma and 83 with squamous cell carcinoma) [36]. Of the over 60 microRNAs analyzed, microRNA-126 was the most predictive for outcome, with low expression indicating a poor prognosis. This result was confirmed in a meta-analysis of over 1000 patients conducted by Sun and colleagues [37]. Zhang et al., analyzing the expression of microRNA-17-5p in patients with non-small cell lung cancer compared to a healthy control group, found a significant increase in miR-17-5p. The area under the curve (AUC) for this microRNA obtained by the authors was 74.6%. Combining the determination of microRNA-17-5p with three recognized serological biomarkers for the diagnosis of NSCLC, such as SCCA, CEA, and CYFRA21-1, resulted in the AUC value increasing to over 84% [38]. MicroRNA-21 expression levels increase progressively with advancing TNM stage in NSCLC. Studies have shown significantly higher microRNA-21 expression in stage III–IV tumors compared to stage I–II. This upregulation is linked to tumor growth, invasion, metastasis, and chemoresistance. Higher microRNA-21 expression is also associated with lymph node metastasis (N stage) and distant metastasis (M stage). It promotes epithelial-to-mesenchymal transition, aiding metastatic potential. Elevated microRNA-21 levels in tumors or serum correlate with poor overall survival (OS) and shorter disease-free survival (DFS). Grimolizzi et al. obtained interesting results by examining the levels of microRNA-126 in the serum of patients with early non-small cell lung cancer and the control group. They found comparable values in the early stages of cancer, whereas the levels of this microRNA in the serum of patients with advanced NSCLC were significantly reduced [39]. In turn, Wu et al. found significant increases in serum levels of microRNA-21-5p, microRNA-141-3p, microRNA-222-3p, and microRNA-486-5p, as well as microRNA-146a-5p and microRNA-126-3p, in patients with early non-small cell lung cancer. Combining these six microRNAs can provide effective diagnostic results for patients with early NSCLC. The AUC of these microRNAs can reach up to 0.960, with a sensitivity of 85.42% and a specificity of 92.50% [40]. Moreover, Sun et al. reported that the expression of serum microRNA-106b in lung cancer patients was higher than that in healthy individuals, and the level of microRNA-106b correlated with TNM stage and lymph node metastasis. The content of this microRNA in the cell line is very high, and it may enhance the ability of lung cancer cells to migrate and invade. It may also increase the expression of metastasis-related proteins (e.g., metalloproteinases MMP-2 and MMP-9) in the cell line [41].

The gold standard for the quantitative detection of low levels of microRNAs with high sensitivity and specificity is quantitative real-time reverse transcription polymerase chain reaction (qRT-PCR). Another method for rapid and sensitive analysis of microRNA expression based on isothermal rolling circle amplification is the bioluminescence assay. Recently, hybridization-based methods, such as in situ hybridization with locked nucleic acid probes, have been refined and are widely used to simultaneously screen large numbers of microRNAs. Deep sequencing methods based on next-generation sequencers are considered a very good method as they can process millions of sequence reads in parallel in a relatively short time, allowing the detection of most microRNAs. Circulating microRNAs found in various body fluids are also frequently studied using high-throughput profiling techniques, sequencing, and microRNA microarrays combined with qRT-PCR [42].

Unfortunately, only a few significant studies have reported the use of microRNAs in clinical diagnosis or therapy. However, it can be concluded that microRNAs are a promising biomarker for non-small cell lung cancer. The mean Cq values of many microRNAs remained unchanged from 0 to 24 h when serum or plasma were stored on ice. Minimal changes were also observed in the Cq values over 24 h in serum/plasma at room temperature. MicroRNA sequencing detected approximately ~650 different microRNA signals in plasma, with over 99% of the microRNA profile remaining unchanged even when blood draw tubes were left at room temperature for 6 h prior to processing. Therefore, further research is needed on the use of microRNAs in the diagnosis and prognosis of this cancer. It is highly hoped that further work will enable the practical application of microRNAs in patients in the near future.

## 5. Circulating Cell-Free DNA and Circulating Tumor DNA

Genetic alterations caused by tumor development can be detected using circulating cell-free DNA, fragmented DNA chains that freely circulate in the blood and are shed from tumor tissue as a result of processes occurring in tumor cells such as apoptosis, necrosis, and secretions. Genomic cfDNA is made up of long DNA fragments (>500 bp). Under physiological conditions, the amount of cfDNA in the plasma is not high because it is cleared by phagocytes. In people without cancer, cfDNA is primarily derived from genomic DNA released during inflammation or cell apoptosis. Higher levels of ctDNA have been demonstrated in the bloodstream in advanced solid tumors, which have been analyzed in detail [43]. On the other hand, cfDNA allows for non-invasive diagnostic tests in personalized medicine due to the fact that it provides the same molecular information as invasive tumor biopsies. Proper treatment of patients with advanced NSCLC currently requires the evaluation of specific predictive biomarkers according to the molecular characteristics of the disease. Sometimes, the availability of tumor tissue is too limited, making molecular profiling difficult, so cfDNA testing can be used as a surrogate method [44]. The detection of somatic changes in cfDNA depends on the amount of this biomarker and the quality of its sequencing methods. cfDNA dynamically demonstrates tumor development and provides data on the specific mechanisms of primary tumor genome mutations and therapy resistance. cfDNA has been shown to be detected with a sensitivity of over 50% in early-stage NSCLC, suggesting that cfDNA may be a marker in the diagnosis and prognosis of NSCLC. Although the amount of cfDNA in the plasma of these people is low in early-stage NSCLC, the use of new, more sensitive and specific techniques, such as digital polymerase chain reaction and next-generation sequencing (NGS), allows it to be used to detect genetic changes associated with the presence of cancer [45]. Accurate cfDNA analysis detects tumor progression and changes in drug resistance early in the disease process. This data provides more accurate information at the earliest possible stage and increases the effectiveness of therapy. High cfDNA concentrations in blood are also associated with poorer survival; therefore, ctDNA may be a useful factor in estimating prognosis. Furthermore, a stable cfDNA level after surgical treatment for early-stage non-small cell lung cancer may indicate disease recurrence and enable the identification of patients for further intervention [46].

Rapid technical progress has made the identification of low-frequency changes in cfDNA possible. Such platforms include qPCR, digital polymerase chain reaction (dPCR), amplification and magnetism, and NGS. The sensitivity of these methods ranges from 15 to 0.01%, and one of the main drawbacks in their clinical use is insufficient standardization [47]. It is important to note that due to the short mean half-life of ctDNA (~1.5–2 h), plasma should be separated and frozen within 3 h of collection. However, this very short half-life of ctDNA makes it an ideal biomarker for monitoring non-small cell lung cancer [48].

## 6. Tumor-Educated Platelets

Platelets can play a significant role in carcinogenesis, influencing, among other things, tumor evasion and metastasis. Activated platelets secrete various factors and substances that interact with factors released by tumor cells. This interaction between the tumor, the tumor microenvironment, and platelets is responsible for the formation of tumor-derived platelets [49]. This process induces specific pre-mRNA splicing, and platelets can ingest circulating RNA. Platelets change their RNA content in response to tumor-related signals, but generally speaking, TEP-enriched RNA transcripts are ontologically linked to platelet activity and platelet vesicles. Platelet RNA profiles distinguish patients with localized and metastatic cancer from healthy individuals with an accuracy of 80–97% [50]. However, extensive large-scale validation demonstrated a decrease in the sensitivity of TEP-based tests in non-small cell lung cancer (50% for stage I, 70% for stage II, 63% for stage III, and 77% for stage IV), with a specificity of 99%. Unfortunately, the specificity of the test decreased below 80% when the control group included patients with benign tumors, inflammatory diseases, and cardiovascular diseases [51]. Best et al. conducted TEP studies on 779 patients with non-small cell lung cancer and 339 cancer-free individuals and found an accuracy of ~88%, AUC ~0.94 (95% CI ~0.92–0.96) for late-stage NSCLC and an accuracy of ~81%, AUC ~0.89 (95% CI ~0.83–0.95) for early-stage NSCLC [52].

TEPs sequester tumor-derived EML4–ALK fusion transcripts, which were reduced after successful crizotinib therapy in a patient with NSCL. Platelet survival is approximately ten days long, and the transcript released from the tumor can collect in TEPs and be insensitive to action of plasma ribonucleases. Therefore, TEP RNA analysis allows for the detection of NSCLC with higher sensitivity and thus monitoring treatment efficacy [53]. Importantly, TEP testing appears to be possible using only small amounts of platelet RNA (up to 500 pg) extracted from routinely collected volumes of plasma samples. TEPs can sequester over 5500 RNA markers and are promising agents for the diagnosis of cancers, including non-small cell lung cancer. However, the mechanism of platelet formation in tumors remains unclear. The clinical usefulness of tumor-educated platelets as biomarkers requires further research, and algorithms based on TEPs still need to be refined.

## 7. Circulating Extracellular Vesicles

Extracellular vesicles (EVs) are small, typically being 40–150 nm in diameter (with an average diameter of approximately 100 nm); they are protected by two lipid layers from degradation by various enzymes. They are formed during multi-step endocytosis in various cell types, both non-cancerous and cancerous, and they are released into the extracellular space. EVs transport different cellular substances, e.g., lipids, proteins, and nucleic acids (DNA, messenger RNA, and non-coding RNA) [54]. Extracellular vesicles participate in intercellular communication and alter the molecular bioactivity of recipient cells by releasing a range of biological factors. Consequently, cancer cells release exosomes, which contain cancer-specific biomarkers that allow for the detection of primary tumor characteristics. EVs released by cancer cells can be taken up by neighboring stromal cells, leading to changes in the cellular program [55]. The number of exosomes in cancer patients has been shown to be higher than in healthy individuals [56]. There is growing evidence that exosomes play a key role in carcinogenesis, cancer progression, and metastasis of many cancers, including non-small cell lung cancer [57,58]. Wang et al. identified specific exosomal protein markers in the plasma of patients with metastatic non-small cell lung cancer. Lipopolysaccharide-binding proteins found in exosomes have been shown to be very effective in differentiating patients with and without metastases from NSCLC. The sensitivity of the tests was 83.1%, and the specificity was 67% [59]. In turn, Jakobsen et al. studied EVs using an antibody-based EV array. They detected the presence of exosomes displaying surface markers (75% sensitivity and 76% specificity). Based on this, they were able to classify patients with lung cancer with an accuracy of over 75% [60]. Nucleic acid analysis in EVs has proven to be a more effective method for detecting mutations than cfDNA/ctDNA. Thakur et al. demonstrated that tumor-derived exosomes contain double-stranded DNA, which reflects the mutation profile of the primary tumor cells and their entire genome [61]. Therefore, EVs hold great promise as biomarkers for early detection and monitoring of non-small cell lung cancer through analysis of nucleic acids and other markers.

Various methods are used to isolate EVs, including ultracentrifugation combined with a sucrose gradient and isolation using immunobeads, such as magnetically activated cell sorting (MACS). EVs can also be analyzed using transmission electron microscopy (TEM) or nanoparticle tracking analysis (NTA). The presence of numerous membrane-associated proteins, e.g., ICAM-1 or integrins, can also be used, which are identified using flow cytometry or Western blot, quantitative RT-PCR, and nucleic acid sequencing [62].

## 8. Metabolomic and Proteomic Markers

In various cancers, changes in cell metabolism occur, initiating or promoting carcinogenesis or supporting intense proliferation [63]. This specific dysregulation of metabolic pathways, depending on the tumor, may be helpful in cancer diagnosis with liquid biopsy. Modern detection methods, such as liquid chromatography combined with mass spectroscopy (LC-MS), allow the examination of plasma for a wide range of metabolites [64].

Metabolic changes in serum were observed in patients with early-stage NSCLC; there were increased concentrations of ketone bodies and lactic acid and decreased concentrations of lipids, glucose, choline-phospholipid metabolites, betaine, and trimethylamine oxide. Compared to the control group, the concentrations of many amino acids (e.g., glutamine, asparagine, glutamate, aspartate, leucine, tyrosine, cysteine, and isoleucine) were elevated, but the concentrations of tryptophan and methionine were decreased [65]. The dependence of individual tumors on various metabolites may constitute a potential therapeutic target. Non-small cell lung cancer with an activating mutation of Nrf2, a transcription factor, is glutamine-dependent, suggesting the use of glutaminase inhibitors or G6PD inhibitors in therapy [66]. Therefore, plasma metabolomics demonstrates the clinical potential of effectively using liquid biopsy for NSCLC detection and prediction of therapeutic benefit. However, these methods require large-scale validation before their clinical application can be considered significant [67]. Standardization of metabolite detection methods, especially those using liquid chromatography–mass spectrometry (LC-MS), is crucial for the reproducibility and comparability of results across laboratories and studies. To achieve this, adherence to the Metabolomics Standards Initiative (MSI) guidelines for metadata and data reporting is essential, as well as accurate reporting of all LC-MS parameters: column type, mobile phase composition, gradients, and MS settings (Table 4).

Proteomic studies are attracting increasing interest in the diagnosis of NSCLC. Studying various proteins provides data on molecular interactions and signaling pathways, enabling the identification of biomarkers using mass spectrometry [68]. Proteomic biomarkers can be studied in various body fluids such as plasma, serum, urine, saliva, and sputum [69]. They can also be detected in exhaled breath condensate, bronchoalveolar lavage fluid, and pleural fluid, which is very useful in the diagnosis of non-small cell lung cancer [70]. Zhang et al., based on the study of five biomarkers (ferritin light chain, mitogen-activated protein kinase 1 interacting protein 1-like, fibrinogen beta chain, RAB33B, and RAB15, a member of the RAS oncogene family), differentiated lung cancer patients from healthy individuals. The parameters were determined in urine samples [71]. Commonly used biomarkers of lung cancer tested individually or in panels of several combined biomarkers include the well-known cytokeratin fragment 19 (CYFRA 21-1), carcinoembryonic antigen (CEA), squamous cell carcinoma antigen (SCCA), and carbohydrate antigen 125 (CA125) [72,73]. Other researchers propose various combinations of previously used markers with new biomarkers, such as tumor/testis antigen 1B, prolactin, retinol-binding protein, thymidine kinase 1, 1-antitrypsin (ATT), neuron-specific enolase, or the autoantibodies A1-Ab and β-enolase-Ab [73,74,75,76]. All the above studies show that the sensitivity and specificity of the tests increase with the expansion of the biomarker panel compared to the effectiveness of a single marker. Furthermore, testing for autoantibodies in conjunction with commonly used biomarkers may also increase the usefulness of these biomarkers in the early diagnosis of NSCLC, as autoantibodies arise early in the tumor progression process and can be detected in serum more quickly than tumor-associated antigens [77].

Broadband proteomic analyzes are typically limited to scientific applications due to the complexity of interpreting the large amounts of data generated by high-throughput mass spectrometry methods. Although several of the proposed new proteomic biomarkers remain controversial due to discrepancies between individual studies, they demonstrate significant potential and may become significant over time, as the level of validation studies increases [78,79].

## 9. Conclusions and Future Perspectives

The lack of sensitive and effective biomarkers for cancer diagnosis and prognosis is the main cause of the poor survival rate of cancer patients. Liquid biopsy, as a minimally invasive method unlike traditional tissue biopsy, may make significant progress in the diagnosis and treatment of NSCLC [80]. New technologies, including mutation analysis supported by machine learning and multimodal liquid biopsy, allowing for the examination of microRNAs, circulating cell-free DNA, TEPs, CTCs, and circulating extracellular vesicles constitute promising directions for future research. These new biomarkers are being intensively studied in patients with NSCLC because they have been proven to provide dynamic molecular data on changes in the course of the disease and can be used for non-invasive monitoring of therapy. The prevailing view is that metastases and tumors are highly heterogeneous, and therefore each stage should be treated as a distinct state. For example, cell-free DNA from plasma has been shown to be suitable for monitoring treatment response and for early detection of acquired resistance mechanisms (e.g., T790M in non-small cell lung cancer) [81]. Also, in the case of examining circulating tumor cells in lung cancer, there is growing interest in developing new techniques for molecular characterization of CTCs, which is strongly related to assessing prognosis. It is not fully understood whether CTCs are the sole cause of metastasis, but their number has been shown to correlate with an unfavorable prognosis in NSCLC and other tumor types. However, clinical validation of these results remains necessary, particularly in non-small cell lung cancer [82]. Additionally, other circulating markers—e.g., extracellular vesicles, which are found in many body fluids (e.g., blood and plasma)—contain tumor-derived genetic material. They are being intensively analyzed for their clinical utility in the diagnosis, treatment monitoring, and prognosis of NSCLC [83,84]. This confirms that molecular biomarkers can provide new and non-invasive sources of material that can be routinely used in predictive biomarker testing for targeted therapies. However, despite a number of studies confirming the feasibility of liquid biopsy using new biomarkers, at the present time, there is no universal consensus on the criteria for using these circulating markers as prognostic biomarkers in non-small cell lung cancer. It should be noted that there are limitations such as lack of consistency, problems with standardizing sample types and preparation methods, the choice of isolation method, sample heterogeneity, and variability in marker expression (for example, CTCs undergoing EMT do not express epithelial markers), reproducibility, selectivity, and sensitivity [85]. Additionally, technology dependent on the use of appropriate sampling tubes, volumes, and subsequent analysis methods should be standardized, as these aspects can affect data quality and contribute to discrepancies and statistical errors. Key technologies at this time are

- ctDNA/cfDNA;
NGS panels (e.g., EGFR, ALK, KRAS, BRAF);
Digital PCR (dPCR);
Methylation-based liquid biopsy;
Exosome and miRNA-based assays (emerging);
AI/ML algorithms for data interpretation.

Torga and Pienta validated liquid biopsy of identical samples in two different laboratories for independent testing and demonstrated complete mutation matching in only 7.5% of cases. These results demonstrate the need for standardization and system development and the importance of multicenter and extensive clinical validation of each method before its use in routine clinical practice. Furthermore, standardized blood and sample collection protocols are required to minimize technical errors (Table 5) [86].

Despite these limitations, it is expected that the possibility of using liquid biopsy in routine clinical practice will certainly increase in the future. Liquid biopsy data, along with imaging analysis replacing invasive tissue biopsies, are likely to influence therapeutic decisions. So, by combining precision diagnostics with personalized therapy, liquid biopsy has the potential to transform cancer care, improving patient outcomes around the world. In summary, this review supports the continued development of liquid biopsy in routine clinical practice for patients with non-small cell lung cancer. Each of the biomarkers discussed in this review, including miRNAs, cfDNAs, CTCs, EVs and TEPs, have significant potential in cancer diagnosis and monitoring. Although significant challenges remain, the potential impact on individual therapeutic strategies, early detection of tumor recurrence, and greater treatment efficacy make liquid biopsy a valuable and improved tool in oncology. The timeline of liquid biopsy milestones in the detection of NSCLC is as follows:

1990s—Early Foundations
1994: Discovery of circulating tumor DNA (ctDNA) in blood.
2000s—Conceptualization and Early Studies
2008: First research indicating cfDNA could be used to detect EGFR mutations non-invasively in NSCLC patients.
2010–2013—Technological Advances
2010: Rise of next-generation sequencing (NGS) enables more precise mutation detection from small DNA fragments.
2012: Early clinical studies confirm feasibility of detecting EGFR mutations in plasma of NSCLC patients.
2014–2016—Clinical Validation and FDA Recognition
2014: First large-scale validation studies of ctDNA for EGFR mutations in NSCLC.
2016: FDA approves the cobas^®^ EGFR Mutation Test v2 (Roche), the
first liquid biopsy test approved for NSCLC.
2017–2019—Expanded Panels and MRD
2017: Introduction of multi-gene liquid biopsy panels (e.g., Guardant360, FoundationACT) for broader mutation profiling in NSCLC.
2019: Liquid biopsy explored for minimal residual disease (MRD) detection and recurrence monitoring.
2020: NCCN guidelines include liquid biopsy as an alternative when tissue is insufficient or unavailable.
2021–2022: Studies show ctDNA use in early-stage NSCLC for MRD and early relapse prediction.
2023–2025—Early Detection and AI Integration
Growing focus on stage I/II NSCLC detection via ultrasensitive ctDNA and methylation assays.
2024–2025 (projected milestones):
AI/ML-enhanced ctDNA interpretation for early diagnosis and patient stratification.

Using new techniques such as artificial intelligence or single-cell RNA sequencing could address long-standing challenges, such as discovering diagnostic and prognostic biomarkers, to help physicians more precisely select medications. These data layers integrate advanced computational methods to provide a comprehensive overview of the cancer process. Rethinking resistance to checkpoint inhibitors (e.g., PD-L1) or targeted therapies using new methods could help select only patients who would benefit from these therapies in the future. Robust, high-throughput clinical outcomes are crucial for clinical biomarker discovery. Therefore, international collaboration with large samples covering a wide range of cancers and sharing treatment data is essential to develop more accurate biomarkers to predict treatment response in lung cancer patients. Early detection and appropriate treatment prediction may ultimately help clinicians improve survival rates for these patients. Below, we present a 3-year roadmap for multi-omics biomarker validation (Table 6).

**Year 1: Foundation and Discovery**

1. Cohort Design and Data Collection
  - Define clinical questions and endpoints.
  Assemble cohorts: Design multi-center prospective cohorts or leverage existing biobanks with matched clinical data. This includes
  Adequate sample size for statistical power;
  Diverse demographics to ensure generalizability;
  Standardize protocols which harmonize sample collection, processing, and storage across sites to reduce batch effects;
  Multi-omics profiling;
  A data integration framework that sets up secure databases and pipelines for multi-omics data management and preprocessing.
2. AI-Driven Biomarker Discovery
  - Exploratory analysis;
  Feature selection;
  Multi-modal integration;
  Model interpretability;
  Initial validation: Cross-validation within cohorts and retrospective validation on independent datasets.

**Year 2: Validation and Optimization**

1. Prospective Validation and Refinement
  - Independent cohort testing;
  Longitudinal validation;
  Analytical validation.
2. AI Model Refinement and Clinical Contextualization
  - Refine predictive models;
  Robustness testing through stress-testing models against confounders and missing data;
  Clinical usability studies.
3. Pilot Integration into Clinical Workflows
  - Integration of biomarker-based AI models into pilot clinical decision support tools with user-friendly interfaces;
  Workflow integration;
  User training;
  Pilot testing by conducting small-scale implementation studies to assess feasibility, clinician acceptance, and preliminary impact on decision-making.

**Year 3: Clinical Deployment and Impact Assessment**

1. Clinical Validation and Regulatory Submission
  - Large-scale clinical trials;
  Health economics analysis through cost–benefit evaluation and reimbursement pathways.
2. Full-scale Integration into Clinical Decision Support Systems
  - System integration;
  Real-time decision support;
  Continuous learning;
  Multi-stakeholder engagement through collaboration with payers, regulators, and patient advocacy groups to ensure broad adoption.
3. Post-deployment Monitoring and Expansion
  - Performance monitoring;
  Scalability and generalizability;
  Knowledge dissemination.

## Figures and Tables

**Table 1:** Diagnostic performance of liquid biopsy in NSCLC.

Group/Markers	Significance
Circulating tumor cells	Diagnosis, prognostic, monitoring response to treatment
Circulating free microRNAs	Early diagnosis, prognostic, metastasis, monitoring, response to treatment
Circulating free DNA	Diagnosis and prognostic
Tumor-educated platelets	Early diagnosis, monitoring, response to treatment
Circulating extracellular vesicles	Diagnosis, metastasis, response to treatment
Metabolomic markers	Early diagnosis, prediction, response to treatment
Proteomics markers	Early diagnosis, prognostic, monitoring

**Table 2:** Comparison of CellSearch and Microfluidic platforms.

Feature	CellSearch (FDA-Approved)	Microfluidic Platforms (e.g., Celsee, CTC-Chip, Auto-ICell)
Sensitivity	~70% (EpCAM + CTCs)	Up to 94–96%
Specificity	93–100%	100%
Cost	€700–€1500/test	Variable; generally higher due to technical complexity
CTC phenotype	Primarily EpCAM + (epithelial) CTCs	Epithelial and mesenchymal CTCs
Clinical use	Widely used in clinical settings	Emerging; research-focused
Turnaround time	1–3 days	1–3 days
Enrichment strategy	Immunomagnetic (EpCAM-based)	Physical properties, size, deformability
Viability assessment	No	Yes

**Table 3:** Selected microRNAs in NSCLC.

Symbol	Expression	Materials	Biomarker
microRNA-23-5p	Up	Plasma	Prediction of survival
microRNA-32-5p	Down	Whole blood	Diagnosis, prognosis
microRNA-502b-3p	Up	Plasma	Diagnosis, prognosis
microRNA-200c	Up	Whole blood	Early diagnosis, prognosis
microRNA-150-5p	Up	Whole blood	Early detection, monitoring recurrences
microRNA-122-3p	Down	Whole blood	Diagnosis, prognosis
microRNA-492a-3p	Down	Plasma	Diagnosis, prognosis
microRNA-20a	Down	Whole blood	Prediction of survival
microRNA-2223	Down	Whole blood	Diagnosis, Prognosis
microRNA-145	Up	Plasma	Prediction of survival
microRNA-448	Up	Plasma	Diagnosis, prognosis
microRNA-628-3p	Up	Serum	Diagnosis, prognosis
microRNA-210	Up	Plasma	Diagnosis, prognosis
microRNA-29c	Down	Whole blood	Early detection
microRNA-124	Up	Plasma	Diagnosis, prognosis
microRNA-126	Down	Serum	Diagnosis, prognosis, response to treatment
microRNA-17-5p	Up	Serum	Prediction of survival
microRNA-21-5p	Up	Serum	Early detection, prognostic
microRNA-141-3p	Up	Serum	Early detection
microRNA-222-3p	Up	Serum	Early detection
microRNA-486-5p	Down	Serum	Early detection
microRNA-146a-5p	Up	Serum	Early detection
microRNA-126-3p	Down	Serum	Early detection
microRNA-106b	Up	Serum	Monitoring recurrences. metastasis

**Table 4:** Methodological comparison of metabolics platforms.

Platform/Module	Typical Mass Accuracy (or Analogous)	Dynamic Range (Linear, Orders of Magnitude)	QA/QC Measures and Challenges
LC-HRMS (Orbitrap, QTOF)	~1–5 ppm (some <1 ppm for small molecules; ≤10 ppm typical)	~10^4^ to 10^5^ (4–5 orders)	System suitability tests, internal isotope standards, pooled QC every N samples, blanks, retention time monitoring, drift correction, signal normalization, QC of mass error and retention time deviation; intra-batch CV ≤20–30%
GC-MS (high-res and uni-res)	~1–3 ppm(high-res) or 10s of 100s mDa (low-res)	~10^4^ (sometimes up to 10^5^)	Retention index calibrants, test mix injections, blanks, drift monitoring, internal standards, replicate injections, detector linearity checks, carry-over monitoring
Direct infusion/FIA-MS	~1–10 ppm (depends on MS)	~10^3^ to 10^4^ (ion suppression limits range)	Vulnerable to ion suppression/matrix effects; use internal standards, repeated QC injections, drift correction, dilution curves, artifact flagging tools
FT-MS/FT-ICR	<<1 pmm (sub-pmm, sometimes 10s of ppb)	~10^5^ to 10^6^ + (very high)	Requires exceptional stability, regular calibration, lock masses, system suitability, drift monitoring, isotope pattern verification)
NMR (1H, 13C)	Chemical shift reproducibility ~0.001–0.005 ppm; spectral resolution ~0.5–1 Hz	~10^3^ to 10^4^	Calibration (chemical shift references), shimming, temperaturę stability, instrument checks, replicate measurements, quantitation with standards, QC samples, signal-to-noise monitoring
Other/hybrid (ion mobility MS, LC-MS/MS MRM)	Varies; targeted MRM precision high (mDa or better)	Up to 10^5^ or more (targeted)	Calibration curves; low/medium/high-QC samples, reference materials, replicate injections, retention time and transmission monitoring, carry-over checks

**Table 5:** NSCLC biomarker clinical utility matrix.

Biomarker Type	Detection Window	Invasiveness	Cost	Sensitivity	Specificity	Clinical Use Stage
CTCs	Narrow to moderate (detectable in advanced stages)	Low (blood draw)	High	Moderate	High	Limited/research; FDA-approved for prognosis in other cancers (e.g., breast, colon)
cfDNA	Moderate (early to late stages)	Low	Moderate to high	High	Moderate to high	Widely used for EGFR mutation testing; approved in NSCLC (liquid biopsy)
TEPs	Moderate to broad	Low	Moderate	High	High	Experimental/research
EVs	Broad (early detection potential)	Low	Moderate to high	High	High	Experimental/promising for early diagnosis
mRNA	Moderate (can vary by stability)	Low	Moderate	Variable	Variable	Research; mRNA panels under evaluation
Circulating proteins	Broad (many secreted early)	Low	Low to moderate	Moderate	Moderate	Diagnostic panels in use (e.g., CYFRA 21-1, CEA); not specific alone
Circulating metabolites	Moderate (affected by systemic factors)	Low	Low to moderate	Variable	Variable	Research stage; metabolomic signatures under development

**Table 6:** Summary of the 3-year roadmap for multi-omics biomarker validation.

Year	Key Activities	Milestones
**1**	Cohort design, multi-omics data generation, AI discovery	Cohort established, biomarker candidates identified
**2**	Validation in independent cohorts, model refinement, pilot CDSS	Validated biomarkers, prototype CDSS tested
**3**	Clinical trials, regulatory approval, full CDSS integration	Clinical utility proven, CDSS deployed

## Data Availability

No new data were created or analyzed in this study.
